# Circular RNA Protein Tyrosine Kinase 2 Promotes Cell Proliferation, Migration and Suppresses Apoptosis *via* Activating MicroRNA-638 Mediated MEK/ERK, WNT/β-Catenin Signaling Pathways in Multiple Myeloma

**DOI:** 10.3389/fonc.2021.648189

**Published:** 2021-07-28

**Authors:** Fan Zhou, Dongjiao Wang, Nian Zhou, Haimin Chen, Haotian Shi, Rong Peng, Wei Wei, Lixia Wu

**Affiliations:** Department of Hematology and Oncology, Shanghai Jing’an District Zhabei Central Hospital, Shanghai, China

**Keywords:** circ-PTK2, miR-638, multiple myeloma, MEK&ERK, WNT&β-catenin

## Abstract

Our previous study observed that circular RNA protein tyrosine kinase 2 (circ-PTK2) was upregulated and correlated with worse clinical features and unfavorable prognosis in multiple myeloma (MM) patients. Thus, this study aimed to further characterize the regulatory function of circ-PTK2 on cell malignant activities and its target microRNA-638 (miR-638) as well as downstream MEK/ERK, WNT/β-catenin signaling pathways in MM. The effect of circ-PTK2 on MM cell proliferation, apoptosis, migration, invasion and its potential target miRNAs was assessed by transfecting circ-PTK2 overexpression plasmids into U226 cells and circ-PTK2 knock-down plasmids into LP-1 cells. Furthermore, the interaction between circ-PTK2 and miR-638 mediated MEK/ERK and WNT/β-catenin signaling pathways was validated by rescue experiments. Circ-PTK2 was overexpressed in most MM cell lines compared to normal plasma cells. Overexpressing circ-PTK2 promoted proliferation and migration, inhibited apoptosis in U266 cells, but did not affect cell invasion; knocking down circ-PTK2 achieved opposite effect in LP-1 cells. Besides, circ-PTK2 reversely regulated miR-638 expression but not miR-4690, miR-6724, miR-6749 or miR-6775. The following luciferase reporter assay illustrated the direct bind of circ-PTK2 towards miR-638. In rescue experiments, overexpressing miR-638 suppressed proliferation, migration, while promoted apoptosis in both wild U266 cells and circ-PTK2-overexpressed U266 cells; meanwhile, overexpressing miR-638 also suppressed MEK/ERK and WNT/β-catenin pathways in both wild U266 cells and circ-PTK2-overexpressed U266 cells. Knocking down miR-638 achieved opposite effect in both wild LP-1 cells and circ-PTK2-knocked-down LP-1 cells. In conclusion, circ-PTK2 promotes cell proliferation, migration, suppresses cell apoptosis *via* miR-638 mediated MEK&ERK and WNT&β-catenin signaling pathways in MM.

## Introduction

Multiple myeloma is a bone marrow-originated plasma cell malignancy that accounts for about 13% of hematological malignancies (1, 2). The incidence of MM varies among different ethnic groups being twice as common in the black as in the white, and less common in Asian (3). As the mainstay of MM treatment in the early 2000s, chemotherapy in combination with steroids realize a median survival of 3-5 years, whereas with the development of new agents including novel targeted therapies, immunomodulatory drugs and stem cell transplantation during the past 15 years, the median survival has increased to 7-8 years (2). Although many treatment schemes have been developed, most of MM patients are unable to achieve a true cure because they eventually relapse and seek for salvage treatment (1). What’s more, drug resistance as well as treatment-related toxicity are risk factors hindering the prognosis of MM patients (1). On account of this, researches are dedicating to exploring biological characteristics and pathogenesis of MM, as well as identifying additional new regimens with help from cutting edge molecular technologies.

Circular RNAs (circRNAs) are non-coding RNAs structurally presenting as covalent loops without 5′or 3′ tails, which have been disclosed to regulate tumorigenesis in various types of cancers including in hematological malignancies (4–6). However, limited evidence about the role of circRNAs in MM is available currently. In our previous study we screened the circRNA expression profile in MM and discovered 122 upregulated and 260 downregulated circRNAs in MM patients compared with healthy controls by microarray. Further reverse transcription quantitative polymerase chain reaction (RT-qPCR) validation in larger sample size identified 3 circRNAs (circ-PTK2, circ-RNF217 and circ-AFF2) that were potential biomarkers for MM risk as well as prognosis (7). Notably, one of these candidate circRNAs, circ-PTK2, was upregulated and strongly correlated with worse clinical features and unfavorable prognosis in MM patients. Besides, bioinformatics prediction disclosed that circ-PTK2 involved in MM pathogenesis by sponging its potential target microRNA (miR) -638, miR-4690, miR-6742, miR-6749 and miR-6775, among which miR-638 was especially known to be of critical value due to its correlation with MM-related signaling pathways, WNT/β-catenin pathway and MEK/ERK pathway (7–9). Whereas the molecular mechanism of circ-PTK2 and its potential target miR-638 or the subsequent signaling pathways in pathogenesis of MM is still unknown.

Therefore, in this study, we detected the effect of circ-PTK2 on MM cell proliferation, apoptosis, migration, invasion, then characterized the regulatory function of circ-PTK2 on its target miR-638 mediated MEK/ERK and Wnt/β-catenin signaling pathways in MM.

## Methods

### Cell Culture

Human MM cell lines (including NCI-H929, U266, LP-1 and RPMI-8226) were all purchased from Leibniz Institute DSMZ-German Collection of Microorganisms and Cell Cultures GmbH (DSMZ, Germany). The 293T cells were purchased from American Type Culture Collection (ATCC, USA). The NCI-H929 cells, U266 cells and RPMI-8226 cells were cultured in Roswell Park Memorial Institute (RPMI) 1640 Medium (Gibco, USA) containing 10% fetal bovine serum (FBS) (Gibco, USA). The LP-1 cells were cultured in Iscove’s Modified Dulbecco’s Medium (IMDM) containing 10% FBS (Gibco, USA), and 293T cells were cultured in Dulbecco’s Modified Eagle’s Medium (Gibco, USA) containing 10% FBS (Gibco, USA). All cells were maintained in a humidity atmosphere of 5% CO_2_ at 37°C. Human normal bone marrow mononuclear cells (BMMCs) were purified with CD138-coated magnetic beads (Miltenyi Biotec, Germany) from bone marrow samples donated by health donors. This study was approved by Shanghai Jing’an District Zhabei Central Hospital and conducted by following the guideline of Ethical Guidelines for Human Genome/Gene Research issued by the Chinese Government. Written informed consents were provided by all participants before enrollment.

### Circ-PTK2 Expression in MM Cells

RT-qPCR was completed to determine the expression of circ-PTK2 in human MM cell lines with normal plasma cells (CD138^+^ BMMCs) served as control.

### Lentivirus Construction and Cells Infection

Circ-PTK2 overexpression (OE-circ-PTK2) plasmid and circRNA control overexpression (OE-circ-NC) plasmids were constructed with pLO5-ciR vector (catologue No. GS0107)) by Guangzhou Geneseed Biotech Co., Ltd (Guangzhou, China). The sequence of OE-circ-PTK2 plasmid referred to circBase database (http://www.circbase.org/) with access No. circRNA ID: hsa_circ_0005273. Circ-PTK2 knock-down (KD-circ-PTK2) plasmid and circRNA control knock-down (KD-circ-NC) plasmids were constructed using pGLVU6/Puro vector (catologue No. C06002, Genepharma, China) *via* a third company Hanbio Biotechnology (Shanghai, China). The sequence of KD-circ-NC plasmid was 5’-CACCGAGGAAAGATTTCTGCCCATTCGAAAATGGGCAGAAATCTTTCCTC-3’. After construction, OE-circ-PTK2 plasmid or OE-circ-NC plasmid was co-transfected with pHelper 2.0 (Genechem, China) into 293T cells by Lipofectamine 3000 Reagent (Invitrogen, USA) to construct OE-circ-PTK2 lentivirus or OE-circ-NC lentivirus. Then KD-circ-PTK2 plasmid or KD-circ-NC plasmid was co-transfected with pHelper 2.0 (Genechem, China) into 293T cells by HiPerFect transfection reagent (Qiagen, Germany) to construct KD-circ-PTK2 lentivirus or KD-circ-NC lentivirus. U266 cells were individually infected with OE-circ-PTK2 lentivirus and OE-circ-NC lentivirus with 2 μg/ml polybrene (Sigma, USA) for 24 hour (h) and followed by selection with 2 μg/ml puromycin (Sigma, USA) for 7 days to generate OE-circ-PTK2 U266 cells and OE-circ-NC U266 cells. Meanwhile, U266 cells cultured under normal condition were defined as Blank U266 cells. To generate KD-circ-PTK2 cells and KD-circ-NC cells, LP-1 cells were infected with KD-circ-PTK2 lentivirus and KD-circ-NC lentivirus respectively, followed by selection with puromycin (Sigma, USA) according to the method mentioned above. LP-1 cells cultured under normal condition were named as Blank of LP-1 cells. After the selection, RT-qPCR was carried out to detect the expression of circ-PTK2 in the cells. At 0h, 24h, 48h and 72h, cell proliferation was evaluated by cell counting kit-8 (CCK-8) (Dojindo, Japan) according to the manufacturer’s instructions. Cell apoptosis was determined by Annexin V-FITC Apoptosis Detection Kit (Sigma, USA) in accordance with the protocol of kit. Cell migration and invasion ability was measured with TRANSWELL migration assay and invasion assay. In addition, after the transfections, the U266 cells were treated by 0-16 nM bortezomib, the LP-1 cells were treated by 0-32 nM bortezomib to determine the drug sensitivity to bortezomib.

### Target MicroRNA Prediction and Assessment

In our previous study (7), with the application of miRanda Database, circRNA-miRNA network was plotted, through which miR-638, miR-4690, miR-6742, miR-6749 and miR-6775 were considered as the potential target miRNAs of circ-PTK2. Then, the expressions of miR-638, miR-4690, miR-6742, miR-6749 and miR-6775 in the cells were assessed by RT-qPCR.

### MiR-638 Plasmid Construction and Transfection

MiR-638 overexpression (OE-miR-638) and miRNA control overexpression (OE-miR-NC) plasmids were constructed with pCMV-miR vector by Hanbio Biotechnology (Shanghai, China). MiR-638 knock-down (KD-miR-638) and miRNA control knock-down (KD-miR-NC) plasmids were constructed with pCMV-miR inhibitor vector by Hanbio Biotechnology (Shanghai, China). OE-miR-638 plasmid or OE-miR-NC plasmid was transfected into OE-circ-PTK2 cells or OE-circ-NC cells using HiPerFect transfection reagent (Qiagen, Germany), then the cells were divided into OE-circ-PTK2&OE-miR-638 group, OE-circ-PTK2&OE-miR-NC group, OE-circ-NC&OE-miR-638 group, and OE-circ-NC&OE-miR-NC group, respectively. U266 cells without treatment were regarded as Blank control. KD-miR-638 plasmid or KD-miR-NC plasmid was transfected into KD-circ-PTK2 cells or KD-circ-NC cells with the application of HiPerFect transfection reagent (Qiagen, Germany), then cells were divided into KD-circ-PTK2&KD-miR-638 group, KD-circ-PTK2&KD-miR-NC group, KD-circ-NC&KD-miR-638 group and KD-circ-NC&KD-miR-NC group, accordingly. LP-1 cells without any treatment were served as Blank control. The expressions of miR-638 and circ-PTK2 in the cells were determined by RT-qPCR at 24h after transfection. Cell proliferation, cell apoptosis, cell migration and cell invasion ability were detected by the methods described in ‘*Lentivirus construction and cells infection’* section.

### Pathway Analysis

MiR-638 is reported to directly target WNT/β-catenin (catenin beta 1) pathway and MEK (mitogen-activated protein kinase kinase)/ERK (mitogen-activated protein kinase) in cancers other than MM (10, 11). Furthermore, both of the WNT/β-catenin pathway and MEK/ERK pathway play important roles in the progression of MM (8, 9). Hence, to investigate the regulation of circ-PTK2/miR-638 on WNT/β-catenin pathway and MEK/ERK pathway in MM, the protein expressions of WNT1, β-catenin, MEK1/2, phosphate MEK1/2, ERK1/2 and phosphate ERK1/2 were detected by western blot in the cells at 24h after transfection.

### RT-qPCR

Total RNA was extracted using RNeasy Protect Mini Kit (Qiagen, Germany) referring to the manufactures’ protocol. For detection of miRNAs, RNA was reversely transcribed to cDNAs after extraction. For detection of circRNAs, linear RNA was diminished using RNase R (Epicentre, USA) before the RNAs were reversely transcribed to cDNAs. Reverse transcription was conducted using QuantiNova Reverse Transcription Kit (Qiagen, Germany); then, qPCR was performed using QuantiNova SYBR Green PCR Kit (Qiagen, Germany) according to the manufactures’ protocol with GAPDH as internal reference for circ-PTK2, and U6 as internal reference for miRNAs. The primers used were listed in **Table 1**.

**Table 1:** Primers.

Gene	Forward Primer (5’-3’)	Reverse Primer (5’-3’)
circ-PTK2	GCGTCTAATCCGACAGCAACA	AGAGATGCCTGACCTGGATAGA
miR-638	ACACTCCAGCTGGGAGGGATCGCGGGCGGG	TGTCGTGGAGTCGGCAATTC
miR-4690	ACACTCCAGCTGGGGCAGCCCAGCTGAGGC	TGTCGTGGAGTCGGCAATTC
miR-6724	ACACTCCAGCTGGGCTGGGCCCGCGGCGGG	TGTCGTGGAGTCGGCAATTC
miR-6749	ACACTCCAGCTGGGTCGGGCCTGGGGTTGG	TGTCGTGGAGTCGGCAATTC
miR-6775	ACACTCCAGCTGGGTCGGGGCATGGGGGAG	TGTCGTGGAGTCGGCAATTC
U6	CGCTTCGGCAGCACATATACTA	ATGGAACGCTTCACGAATTTGC
GAPDH	GGAGCGAGATCCCTCCAAAAT	GGCTGTTGTCATACTTCTCATGG

### TRANSWELL Migration Assay and Invasion Assay

To perform TRANSWELL migration assay, cells (1x10^5^) suspended in 200 μl culture medium (Gibco, USA) (without FBS) were added into the upper chamber (Corning, USA) (without Matrigel). And the lower chamber was filled with 500 μl culture medium containing 10% FBS (Gibco, USA). After incubation for 4 h, lower chamber medium was collected and the cells in medium were counted by a flow cytometer (BD, USA). Furthermore, to perform TRANSWELL invasion assay, the method described in previous study was conducted (12, 13). The Matrigel (BD, USA), which was dissolved at 4°C overnight, was diluted with FBS-free medium with a ratio of 1:8. The transwell upper chamber was coated with 100 μl diluted Matrigel at 37°C for 1 h. Then, 4 X10^4^ cells suspended in 200 μl FBS-free medium was added into the upper chamber and 500 μl 10% FBS containing medium was added into the lower chamber. Subsequently, the cells were cultured at 37°C for 48 h. After the incubation, the upper chamber was collected and washed with PBS. The upper chamber was then fixed with 700 μl 4% paraformaldehyde (Sigma, USA) for 20 min at room temperature (RT). After washing with PBS, the upper chamber was stained with 0.1% crystal violet (Sigma, USA) for 20 min at RT. Finally, a cotton swab was used to remove the non-invaded cells. The cells were counted and imaged with an inverted microscope (Olympus, Japan).

### Western Blot

Initially, cells were suspended using RIPA Buffer (Sigma, USA), and then Pierce™ BCA Protein Assay Kit (Thermo, USA) was used for protein qualification after centrifugalizing and supernatant collection. Following that, the proteins were re-suspended and isolated on NuPAGE Bis-Tris Gels 4%-12% (Invitrogen, USA) for electrophoresis. Afterwards, the proteins were transferred to polyvinylidene fluoride membrane (PALL, USA), then underwent incubation with primary antibodies overnight and soon after secondary antibodies for 90 min at room temperature. The proteins were chemiluminescenced by EasyBlot ECL kit (Beyotime, China) and visualized by X-ray film (Kodak, USA). The antibodies used were listed in **Table 2**.

**Table 2:** Antibodies.

Antibody	Company	Dilution
**Primary Antibody**		
Rabbit monoclonal to MEK1/2	CST (USA)	1:1000
Rabbit monoclonal to phospho-MEK1/2 (pMEK1/2)	CST (USA)	1:1000
Rabbit monoclonal ERK1/2	CST (USA)	1:1000
Rabbit monoclonal to phospho-ERK1/2 (pERK1/2)	CST (USA)	1:1000
Rabbit polyclonal to WNT1	Abcam (UK)	1:2000
Rabbit polyclonal to β-catenin	CST (USA)	1:1000
Rabbit monoclonal to GAPDH	CST (USA)	1:1000
**Secondary Antibody**		
Goat Anti-Rabbit IgG-HRP	CST (USA)	1:3000

### Luciferase Reporter Assay

Luciferase reporter assay was carried out using Dual-Luciferase^®^ Reporter (DLR™) Assay System (Promega, USA). Circ-PTK2 wild type (WT) plasmid and circ-PTK2 mutant type (MT) plasmid were constructed with pGL4 vector (Promega, USA). OE-miR-638 plasmid, OE-miR-NC plasmid, circ-PTK2 WT plasmid and circ-PTK2 MT plasmid were co-transfected into 293T cells (ATCC, USA) using HiPerFect transfection reagent (Qiagen, Germany). After 48h of transfection, cells were harvested, lysed and firefly luciferase luminescence was detected according to the manufacturer’s instructions.

### Statistical Analysis

GraphPad Prism Software version 7.0 (GraphPad Software Inc., USA) was used for data analysis and graph plotting. All data were presented as mean and standard deviation. One-way analysis of variance (ANOVA) followed by Dunnett’s or Tukey’s multiple comparisons test was performed to determine the comparison among groups. *P* value less than 0.05 was considered as statistically significant. *P*<0.05, *P*<0.01 and *P*<0.001 were represented as *, **, ***. Non-significant (*P*>0.05) was marked as NS.

## Results

### Expression and Functional Significance of Circ-PTK2 on MM Cell Lines

**Figure 1:**
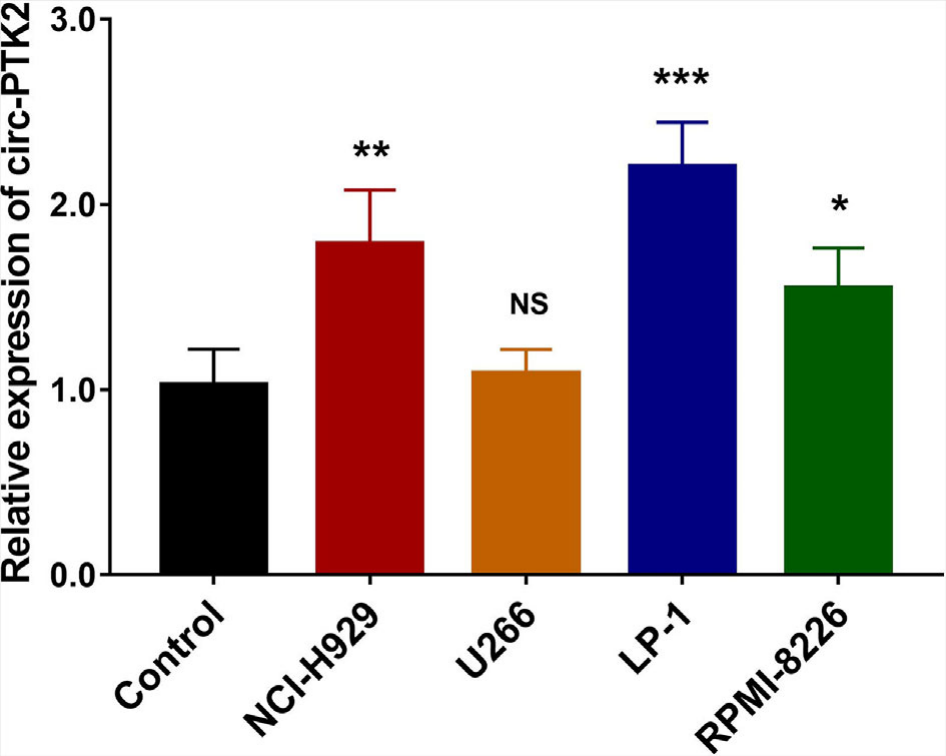
Expression of circ-PTK2 in MM cells. The expression of circ-PTK2 was detected by RT-qPCR in MM cell lines compared with control cells (human CD138^+^ normal bone marrow mononuclear cells purified from health donors). Circ-PTK2, circular RNA protein tyrosine kinase 2; MM, multiple, myeloma; NS, non-significant; RT-qPCR, reverse transcription quantitative polymerase chain reaction; *P < 0.05; **P < 0.01; ***P < 0.001. RNA expression was normalized by 2^-△△Ct^ method. Experiments were performed in triplicates. The comparison was determined by One-way ANOVA followed by Dunnett’s multiple comparison test.

So as to determine whether circ-PTK2 was dysregulated in MM cell lines, RT-qPCR was performed, which observed that expression of Circ-PTK2 was upregulated in NCI-H929 (*P*<0.01), LP-1 (*P*<0.001) and RPM1-8226 cells (*P*<0.05) but similar in U266 cells (*P*>0.05) compared with control (**Figure 1**). Since the lowest circ-PTK2 expression was observed in U266 cell, it was chosen for circ-PTK2 overexpression experiments. Similarly, since the highest circ-PTK2 expression was observed in LP-1 cell, it was used for circ-PTK2 knock-down experiments.

**Figure 2:**
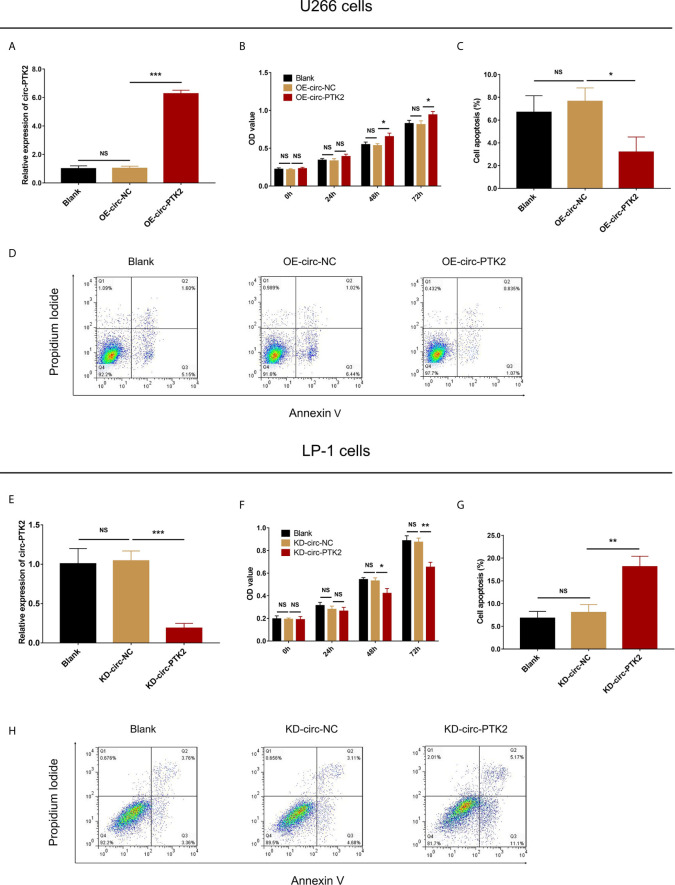
Circ-PTK2 promoted proliferation and suppressed apoptosis of MM cells. The effect of circ-PTK2 overexpression plasmid transfection on circ-PTK2 expression **(A)**, cell proliferation **(B)** and apoptosis **(C, D)** were detected by RT-qPCR, CCK-8 and AV/PI assays in U226 cells. The effect of circ-PTK2 knock-out plasmid transfection on circ-PTK2 expression **(E)**, cell proliferation **(F)** and apoptosis **(G, H)** were detected by RT-qPCR, CCK-8 and AV/PI assays in LP-1 cells. Circ-PTK2, circular RNA protein tyrosine kinase 2; MM, multiple myeloma; OD, optical density; NS, non-significant; OE-circ-NC, circRNA control overexpression; OE-circ-PTK2, circ-PTK2 overexpression; KD-circ-NC, circRNA control knock-down; KD-circ-PTK2, circ-PTK2 knock-down; RT-qPCR, reverse transcription quantitative polymerase chain reaction; CCK-8, counting kit-8; AV/PI, Annexin V/Propidium Iodide; *P < 0.05; **P < 0.01; ***P < 0.001. RNA expression was normalized by 2^-△△Ct^ method. Experiments were performed in triplicates. The comparison was determined by One-way ANOVA followed by Dunnett’s multiple comparison test.

Since circ-PTK2 was greatly higher in MM cell lines, and our previous study observed that circ-PTK2 was related to MM risk, disease features, we further explored its effect on MM cell proliferation and apoptosis *via* CCK-8 assay and AV/PI assay, respectively. In U266 cells, the relative expression of circ-PTK2 was increased by circ-PTK2 overexpression (*P*<0.001) (**Figure 2A**). The cell proliferation by OD value was promoted in OE-circ-PTK2 cells compared with OE-circ-NC cells at 48h (*P*<0.05) and 72h (*P*<0.05) after transfection (**Figure 2B**). Besides, cell apoptosis was suppressed in OE-circ-PTK2 cells compared with OE-circ-NC (*P*<0.05) (**Figures 2C, D** **)**. In LP-1 cells, the relative expression of circ-PTK2 was reduced by circ-PTK2 knock-down (*P*<0.001) (**Figure 2E**). The cell proliferation by OD value was decreased in KD-circ-PTK2 cells compared with KD-circ-NC cells at 48h (*P*<0.05) and 72h (*P*<0.01) after transfection (**Figure 2F**). Besides, cell apoptosis was increased in KD-circ-PTK2 cells compared with KD-circ-NC cells (*P*<0.01) (**Figures 2G, H** **)**.

**Figure 3:**
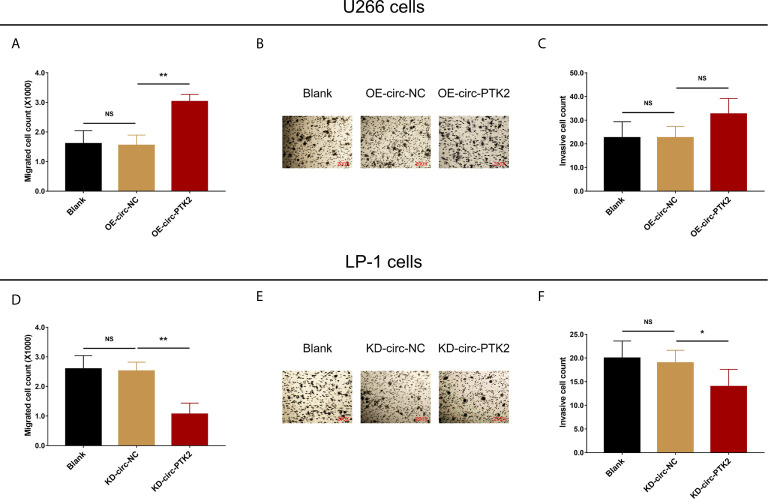
Circ-PTK2 promoted migration but not invasion of MM cells. The effect of circ-PTK2 overexpression plasmid transfection on cell migration **(A)** and invasion **(B, C)** were detected by Transwell migration and invasion assays in U226 cells. The effect of circ-PTK2 knock-out plasmid transfection on cell migration **(D)** and invasion **(E, F)** were detected by Transwell migration and invasion assays in LP-1 cells. Circ-PTK2, circular RNA protein tyrosine kinase 2; MM, multiple myeloma; NS, non-significant; OE-circ-NC, circRNA control overexpression; OE-circ-PTK2, circ-PTK2 overexpression; KD-circ-NC, circRNA control knock-down; KD-circ-PTK2, circ-PTK2 knock-down; *P < 0.05; **P < 0.01. Experiments were performed in triplicates. The comparison was determined by One-way ANOVA followed by Dunnett’s multiple comparison test.

After discovering that circ-PTK2 regulated MM cell proliferation and apoptosis, we further investigated whether it modified MM cell mobility as well *via* Transwell migration and invasion assays. In U266 cells, cell migration was facilitated in OE-circ-PTK2 cells compared with OE-circ-NC cells (*P*<0.01) (**Figure 3A**), whereas cell invasion was similar between the two groups (*P*>0.05) (**Figures 3B, C** **)**. In LP-1 cells, cell migration was attenuated in KD-circ-PTK2 cells compared with KD-circ-NC cells (*P*<0.01) (**Figure 3D**), and cell invasion was also reduced in KD-circ-PTK2 cells compared with KD-circ-NC cells as well (*P*<0.05) (**Figures 3E, F** **)**.

### The Effect of Circ-PTK2 on Bortezomib Sensitivity

Due to that our previous study also observed that circ-PTK2 correlated with treatment response and survival in MM patients, so the effect of circ-PTK2 on bortezomib sensitivity was further evaluated. Relative cell viability was higher in OE-circ-PTK2 cells compared with OE-circ-NC cells under 4-8 nM bortezomib treatment in U266 cells (*P*<0.05) (**Supplementary Figure 1A**). Meanwhile, relative cell viability was lower in KD-circ-PTK2 cells compared with KD-circ-NC cells under 4-32 nM bortezomib treatment in LP-1 cells (*P*<0.05) (**Supplementary Figure 1B**). These data indicated that circ-PTK2 repressed bortezomib sensitivity to some extent.

### The Effect of Circ-PTK2 on the Predicted Target MiRNAs in MM Cells

**Figure 4:**
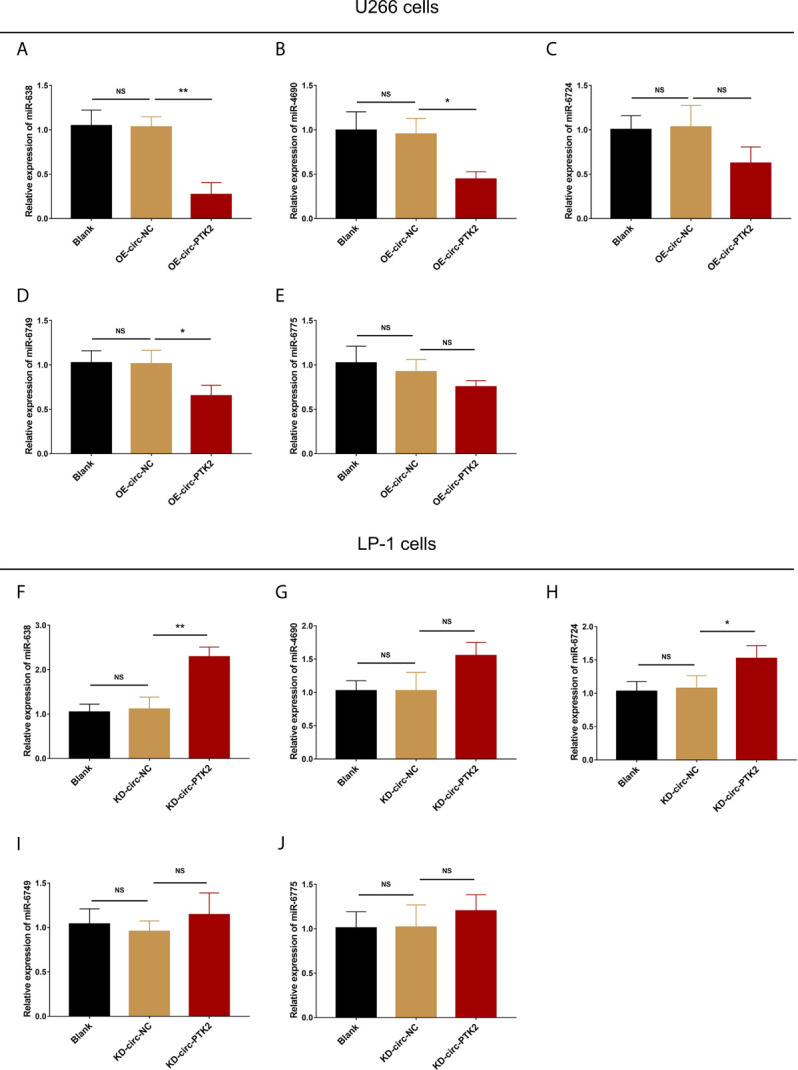
Circ-PTK2 reversely regulated miR-638. The effect of circ-PTK2 overexpression plasmid transfection on miR-638 **(A)**, miR-4690 **(B)**, miR-6724 **(C)**, miR-6749 **(D)** and miR-6775 **(E)** was detected by RT-qPCR in U226 cells. The effect of circ-PTK2 knock-out plasmid transfection on miR-638 **(F)**, miR-4690 **(G)**, miR-6724 **(H)**, miR-6749 **(I)** and miR-6775 **(J)** was detected by RT-qPCR in LP-1 cells. Circ-PTK2, circular RNA protein tyrosine kinase 2; miR, micro RNA; NS, non-significant; RT-qPCR, reverse transcription quantitative polymerase chain reaction; OE-circ-NC, circRNA control overexpression; OE-circ-PTK2, circ-PTK2 overexpression; KD-circ-NC, circRNA control knock-down; KD-circ-PTK2, circ-PTK2 knock-down; *P < 0.05; **P < 0.01. RNA expression was normalized by 2^-△△Ct^ method. Experiments were performed in triplicates. The comparison was determined by One-way ANOVA followed by Dunnett’s multiple comparison test.

**Figure 5:**
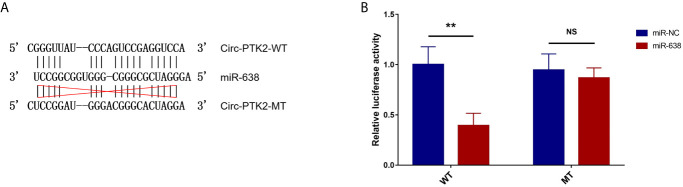
Luciferase reporter assay. The related sequences of circ-PTK2 and miR-638 according to the potential binding site **(A)**. Comparison of relative luciferase activity between miR-638 and miR-NC circ-PTK2-WT cells; comparison of relative luciferase activity between miR-638 and miR-NC circ-PTK2-MT cells **(B)**. Circ-PTK2, circular RNA protein tyrosine kinase 2; miR-638, micro RNA 638; WT, wild type; MT, mutant type; NC, normal control; NS, non-significant; **P<0.01. Experiments were performed in triplicates. The comparison was determined by The comparison was determined by One-way ANOVA followed by Tukey’s multiple comparison test.

Subsequently, we aimed to explore the potential mechanism about the regulatory effect of circ-PTK2 in MM, so its potential target miRNAs were detected *via* RT-qPCR. In U266 cells, the expressions of miR-638 (*P*<0.01) (**Figure 4A**), miR-4690 (*P*<0.05) (**Figure 4B**) and miR-6749 (*P*<0.05) (**Figure 4D**) were reduced in OE-circ-PTK2 cells compared with OE-circ-NC cells, however, miR-6724 (*P*>0.05) (**Figure 4C**) and miR-6775 (*P*>0.05) (**Figure 4E**) expressions were not influenced by circ-PTK2 overexpression. In LP-1 cells, the expressions of miR-638 (*P*<0.01) (**Figure 4F**) and miR-6724 (*P*<0.05) (**Figure 4H**) were increased in KD-circ-PTK2 cells compared with KD-circ-NC cells, however, miR-4690 (*P*>0.05) (**Figure 4G**), miR-6749 (*P*>0.05) (**Figure 4I**) and miR-6775 (*P*>0.05) (**Figure 4J**) expressions were not influenced by circ-PTK2 knock-down. As seen that miR-638 was reversely regulated by both circ-PTK2 overexpression and knock-down, and miR-638 involved in MM-related pathways such as MEK&ERK and WNT&β-catenin signaling pathways, we further performed luciferase assay which validated the direct biding between circ-PTK2 and miR-638 in MM (**Figures 5A, B** **)**. Then, rescue experiments were performed to explore how the interaction between circ-PTK2 and miR-638 regulated cell activities and MEK&ERK and WNT&β-catenin signaling pathways.

### The Effect of MiR-638 and Circ-PTK2 on MM Cell Proliferation and Apoptosis

**Figure 6:**
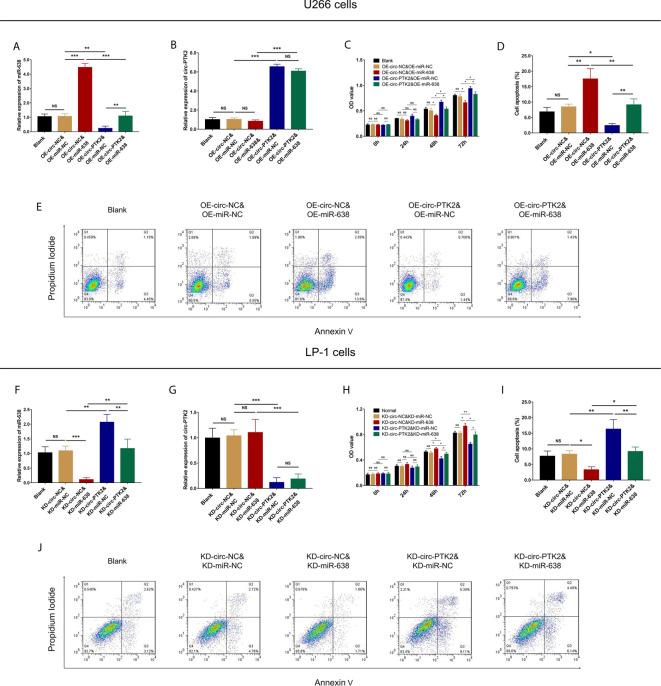
Circ-PTK2 affected cell proliferation and apoptosis *via* regulating miR-638. Comparison of miR-638 expression **(A)**, circ-PTK2 expression **(B)**, cell proliferation **(C)** and cell apoptosis **(D, E)** detected by RT-qPCR, CCK-8 and AV/PI assays among different groups of U266 cells. Comparison of miR-638 expression **(F)**, circ-PTK2 expression **(G)**, cell proliferation **(H)** and cell apoptosis **(I, J)** detected by RT-qPCR, CCK-8 and AV/PI assays among different groups of LP-1 cells. Circ-PTK2, circular RNA protein tyrosine kinase 2; miR-638, microRNA 638; OD, optical density; NS, non-significant; RT-qPCR, reverse transcription quantitative polymerase chain reaction; CCK-8, counting kit-8; AV/PI, Annexin V/Propidium Iodide; OE-circ-NC, circRNA control overexpression; OE-circ-PTK2, circ-PTK2 overexpression; KD-circ-NC, circRNA control knock-down; KD-circ-PTK2, circ-PTK2 knock-down; OE-miR-NC, miRNA control overexpression; OE-miR-638, miR-638 overexpression; KD-miR-NC, miRNA control knock-down; KD-miR-638, miR-638 knock-down plasmid; *P < 0.05; **P < 0.01; ***P < 0.001. RNA expression was normalized by 2^-△△Ct^ method. Experiments were performed in triplicates. The comparison was determined by One-way ANOVA followed by Tukey’s multiple comparison test.

In rescue experiments, we firstly investigated the effect of miR-638 and its interaction with circ-PTK2 on regulating MM cell proliferation and apoptosis *via* CCK-8 assay and AV/PI assay. OE-miR-638 increased the expression of miR-638 in both wild U266 cells (*P*<0.001) and OE-circ-PTK2 U266 cells (*P*<0.01) (**Figure 6A**), whereas it had no effect on circ-PTK2 expression (both *P*>0.05) (**Figure 6B**). As for cell proliferation and apoptosis, OE-miR-638 reduced cell proliferation (both *P*<0.05) (**Figure 6C**) but promoted cell apoptosis (both *P*<0.01) (**Figures 6D** **, E**) in both wild U266 cells and OE-circ-PTK2 U266 cells. Oppositely, KD-miR-638 reduced the expression of miR-638 in both wild LP-1 cells (*P*<0.001) and KD-circ-PTK2 LP-1 cells (*P*<0.01) (**Figure 6F**), whereas it did not affect circ-PTK2 expression (both *P*>0.05) (**Figure 6G**). Regarding cell proliferation and apoptosis, KD-miR-638 promoted cell proliferation (both *P*<0.05) (**Figure 6H**) but suppressed cell apoptosis (both *P*<0.01) (**Figures 6I, J**) in both wild LP-1 cells and KD-circ-PTK2 LP-1 cells. These data indicated that miR-638 reduced MM cell proliferation, promoted cell apoptosis; and it attenuated the effect of circ-PTK2 on MM cell proliferation and apoptosis.

### The Effect of MiR-638 and Circ-PTK2 on MM Cell Migration and Invasion

**Figure 7:**
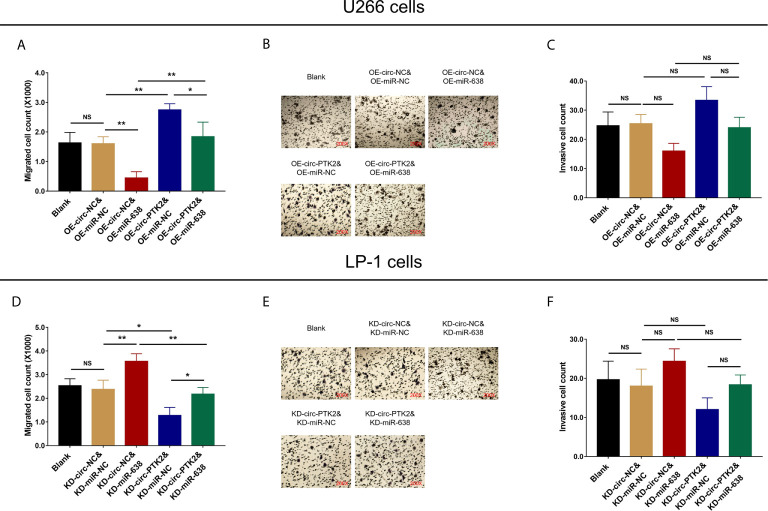
Circ-PTK2 affected cell migration *via* regulating miR-638. Comparison of cell migration **(A)** and invasion **(B, C)** detected by Transwell migration and invasion assays among different groups of U266 cells. Comparison of cell migration **(D)** and invasion **(E, F)** detected by Transwell migration and invasion assays among different groups of LP-1 cells. Circ-PTK2, circular RNA protein tyrosine kinase 2; miR-638, micro RNA 638; NS, non-significant; OE-circ-NC, circRNA control overexpression; OE-circ-PTK2, circ-PTK2 overexpression; KD-circ-NC, circRNA control knock-down; KD-circ-PTK2, circ-PTK2 knock-down; OE-miR-NC, miRNA control overexpression; OE-miR-638, miR-638 overexpression; KD-miR-NC, miRNA control knock-down; KD-miR-638, miR-638 knock-down plasmid; *P<0.05; **P < 0.01. Experiments were performed in triplicates. The comparison was determined by One-way ANOVA followed by Tukey’s multiple comparison test.

In rescue experiments, we secondly explored the effect of miR-638 and its interaction with circ-PTK2 on regulating MM cell migration and invasion *via* Transwell migration and invasion assays. OE-miR-638 reduced cell migration (both *P*<0.05) (**Figure 7A**) but had no effect on cell invasion (both *P*>0.05) (**Figures 7B, C**) in both wild U266 cells and OE-circ-PTK2 U266 cells. Oppositely, KD-miR-638 promoted cell migration (both *P*<0.05) (**Figure 7D**) but not invasion (both *P*>0.05) (**Figures 7E, F**) in both wild LP-1 cells and KD-circ-PTK2 LP-1 cells. These indicated that miR-638 reduced MM cell migration but not invasion; and it attenuated the effect of circ-PTK2 on MM cell migration.

### The Effect of MiR-638 and Circ-PTK2 on MEK&ERK and WNT&β-Catenin Signaling Pathways

**Figure 8:**
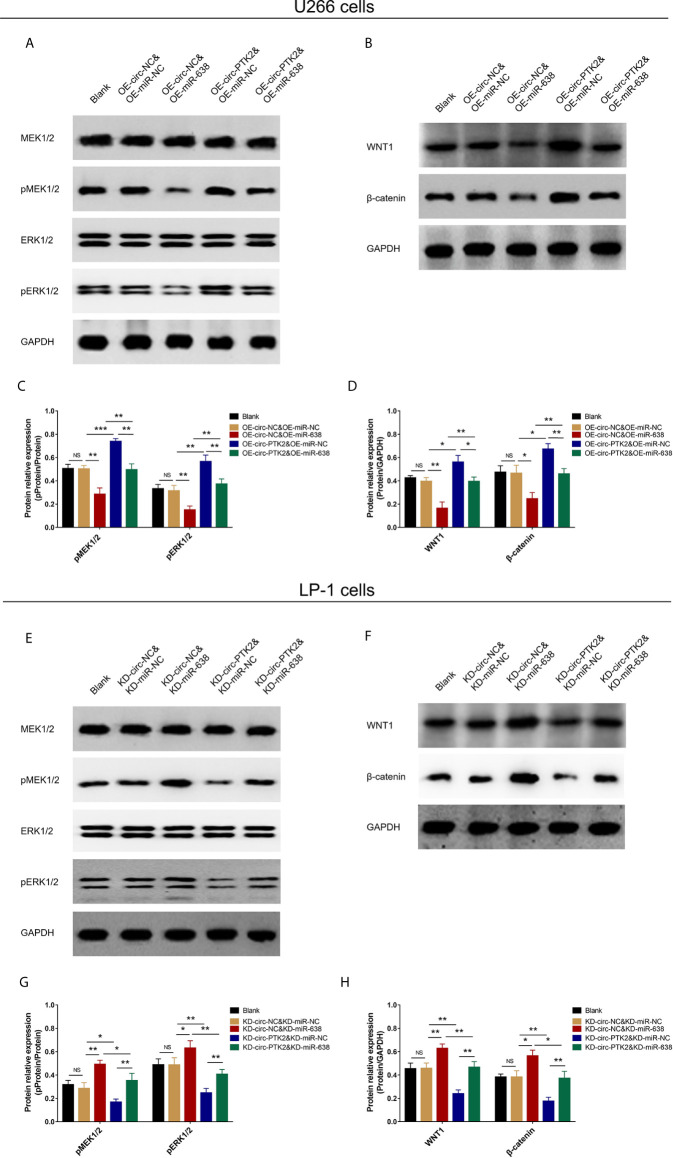
Circ-PTK2 affected Wnt/β-catenin and MEK/ERK signaling pathways *via* regulating miR-638. Comparison of pMEK1/2, pERK1/2, WNT1 and β-catenin protein expressions detected by western blot among different groups of U266 cells **(A–D)**. Comparison of pMEK1/2, pERK1/2, WNT1 and β-catenin protein expressions detected by western blot among different groups of LP-1 cells **(E–H)**. NS, non-significant; OE-circ-NC, circRNA control overexpression; OE-circ-PTK2, circ-PTK2 overexpression; KD-circ-NC, circRNA control knock-down; KD-circ-PTK2, circ-PTK2 knock-down; OE-miR-NC, miRNA control overexpression; OE-miR-638, miR-638 overexpression; KD-miR-NC, miRNA control knock-down; KD-miR-638, miR-638 knock-down plasmid; *P < 0.05; **P < 0.01; ***P < 0.001. Experiments were performed in triplicates. The comparison was determined by One-way ANOVA followed by Tukey’s multiple comparison test.

In rescue experiments, finally, we explored the effect of miR-638 and its interaction with circ-PTK2 on regulating MEK&ERK and WNT&β-catenin signaling pathways in MM *via* western blot. OE-miR-638 tended to reduce pMEK1/2, pERK1/2, WNT1 and β-catenin protein expressions in both wild U266 cells and OE-circ-PTK2 U266 cells (**Figures 8A–D**). Oppositely, KD-miR-638 tended to promote pMEK1/2, pERK1/2, WNT1 and β-catenin protein expressions in both wild LP-1 cells and OE-circ-PTK2 LP-1 cells (**Figures 8E–H**). These indicated that miR-638 suppressed MEK&ERK and WNT&β-catenin signaling pathways; and it attenuated the effect of circ-PTK2 on MEK&ERK and WNT&β-catenin signaling pathways in MM cells.

## Discussion

MM is a highly heterogeneous disease whose treatment outcome varies with chromosomal and genetic differences considerably (2). Despite that novel agents and treatment strategies have improved treatment response and survival in a certain extent, the majority of MM patients do not substantially have curative benefit (1). Hence, new treatment options are always important to optimize the clinical outcomes of MM, especially for those patients with relapse or refractory disease. Based on our previous study, circ-PTK2 was identified to be differentially expressed and related to prognosis in MM clinically (7). As a step further, we investigated the molecular mechanisms of circ-PTK2 in MM pathogenesis in this present study. We observed that (1): circ-PTK2 was expressed in MM cell lines (2); circ-PTK2 facilitated MM cell survival and migration but not cell invasion, and suppressed cell apoptosis (3); circ-PTK2 reversely regulated miR-638 and influenced MM cell activity, activated MEK&ERK and WNT&β-catenin signaling pathway *via* sponging miR-638.

CircRNAs are initially found in RNA virus in 1976 and later in eukaryotes (14, 15). They are formed by the covalent ligating of the 5′ and 3′ ends of exon(s) through back-splicing during pre-RNA splicing (16). CircRNAs contain target sites for miRNAs, and act as sponge to miRNAs in post-transcriptional regulation, which has been reported to modulate cancer progression. For instance, circRNA000190 is a novel circRNA that inhibits the progression of MM *via* targeting miR-767-5p and the downstream MAPK4 (4). The circRNA expression profiles are being investigated in various cancer researches and quite amount of circRNAs are identified as potential cancer biomarkers (7, 17, 18). As for MM, our previous study screened the circRNA expression profile in MM and discovered several circRNAs that correlated with MM risk as well as prognosis. Among the candidate miRNAs, circ-PTK2 was overexpressed in MM patients compared with control, and was correlated with poor clinical characteristics and unfavorable prognosis (7). Besides, the target miRNAs of circ-PTK2 were retrieved from tissue specific circRNA database (http://gb.whu.edu.cn/TSCD/) including miR-638, miR-4690, miR-6742, miR-6749 and miR-6775, however, the detailed mechanism of circ-PTK2 in MM pathogenesis through regulating these target miRNAs was not investigated then. Therefore, in this current experimental study, we initially examined the expression of circ-PTK2 in MM cell lines compared with human normal BMMCs, and found that circ-PTK2 was overexpressed in MM cells. Following that, circ-PTK2 overexpression and knock-down MM cells were constructed respectively, from which circ-PTK2 was shown to promote cell proliferation, migration, and inhibit cell apoptosis, but did not influence cell invasion of MM cells. This was in line with our previous study that circ-PTK2 was correlated with higher MM risk (7), and the reason could be that (1): Circ-PTK2 might act as sponge to miRNAs, which subsequently inhibited the functional regulation of these miRNAs towards cell activity, thereby circ-PTK2 promoted cell proliferation, migration and inhibited cell apoptosis of MM cells. This was further proven in our following rescue experiments and luciferase reporter assay, which showed that circ-PTK2 regulated MM cell activity and activated oncogenic signaling pathways *via* sponging miR-638 (2). It was considered that circRNA could serve as a restoration pool for its parent gene, so we hypothesized that circ-PTK2 also functioned like this: its parent gene PTK2 (also known as FAK) was previously reported to be expressed and regulate MM cell death, invasion, adhesion to bone marrow stromal cells, and drug resistance (19–23). So circ-PTK2 might function in MM *via* regulating PTK2 gene, however, no direct evidence had proved this, which needed further exploration.

MiRNAs, the epigenetic regulator, are non-coding RNAs with 19-25 nucleotides long, and they regulate gene expression *via* degrading the target mRNAs or inhibiting translation (10). In MM, quite a number of miRNAs have been reported to regulate tumorigenesis, invasion as well as chemoresistance (24). For instances, miR-489 suppresses cell growth *via* lactate dehydrogenase isoform A-mediated aerobic glycolysis of MM cells, and miR-182 induces cell adhesion-mediated drug resistance *via* enhancing AKT phosphorylation (24, 25). Known that circRNAs mainly function by sponging target miRNAs in cancers, we hypothesized that circ-PTK2 might sponge its target miRNAs and contribute to the development and progression of MM as well. Enlightened from our previous study, the potential target miRNAs of circ-PTK2 were chosen (including miR-638, miR-4690, miR-6724, miR-6749, and miR-6775) and the influence of circ-PTK2 overexpression/knock-out on expression of these miRNAs was assessed. It was observed that the expression of miR-638 was reduced by circ-PTK2 overexpression and promoted by circ-PTK2 knock-down, whereas for other miRNAs, the influence of circ-PTK2 was not obvious. Furthermore, the direct binding of circ-PTK2 with miR-638 was shown by luciferase reporter assay. Thus, miR-638 was considered as the target miRNA of circ-PTK2 in MM cells. Following that, we conducted rescue experiments and consolidated that circ-PTK2 promoted cell proliferation, migration, and inhibited cell apoptosis *via* reversely regulating miR-638 in MM cells. MiR-638 is known as an important regulator of tumorigenesis as well as leukemogenesis (10, 11, 26, 27). It is downregulated in a variety of solid tumors such as gastric cancer, breast cancer and cervical cancer (10, 11, 16). In detail, miR-638 suppressed tumor cell proliferation and migration in cervical cancer *via* Wnt/β-catenin signaling pathway, as well as in gastric cancer *via* MEK/ERK signaling pathway (10, 11). Furthermore, both WNT/β-catenin pathway and MEK/ERK pathway play important roles in the progression of MM (8, 9). Therefore, we supposed that circ-PTK2 would influence Wnt/β-catenin and MEK/ERK signaling pathways by regulating miR-638. The corresponding rescue experiment showed that circ-PTK2 promoted MEK&ERK and WNT&β-catenin *via* regulating miR-638, which was in line with our hypothesis.

A range of molecular abnormalities and aberrant signaling pathways have been discovered to facilitate the pathogenesis of MM (28, 29). To address, Wnt/β-catenin and MEK/ERK signaling pathways are frequently studied pathways in cancers including MM, of which Wnt/β-catenin pathway is constitutively activated in MM, and it promotes MM cells growth (30). Besides, mutation in Wnt regulatory components strongly promotes the activity of Wnt/β-catenin pathway and plays central role in MM progression (28). As for MEK/ERK, it is part of the RAS signaling cascade, which is the major mutation of MM genetics and it has been identified to regulate cell proliferation and survival in MM cells (29). It is believed that over half of MM patients present MEK/ERK pathway activation, and targeting these pathways have been reported to have therapeutic potential (29). For instance, MEK inhibitor trametinib has achieved promising response in heavily pretreated MM patients (31). Our study disclosed the role of circ-PTK2→miR-638→MEK&ERK/Wnt&β-catenin network in pathogenesis of MM, which added valuable information for both future laboratory research and clinical management of MM.

Although several interesting findings were observed in our present study, some limitations existed (1): A future study was required to validate the findings in an *in vivo* model (2); Wnt reporter assay was not performed in this study, which needed to be further investigated in the future study.

In conclusion, circ-PTK2 promotes MM cell proliferation, migration *via* regulating miR-638 mediated MEK&ERK and Wnt&β-catenin signaling pathways.

## Data Availability Statement

The original contributions presented in the study are included in the article/**Supplementary Material**. Further inquiries can be directed to the corresponding author.

## Ethics Statement

This study was approved by Shanghai Jing’an District Zhabei Central Hospital and conducted by following the guideline of Ethical Guidelines for Human Genome/Gene Research issued by the Chinese Government. The patients/participants provided their written informed consent to participate in this study.

## Author Contributions

FZ conceived of the study. HC, HS, and RP designed the data analysis, with DW and WW conducting the data analysis. NZ and LW wrote the first draft of the original protocol. All authors contributed to the article and approved the submitted version. FZ is the article guarantor.

## Funding

This study was supported by Shanghai Nature Science Foundation (NO. 201840159), Health Research Project of Jing 'an District, Shanghai (NO. 2018QN03) and National Nature Science Foundation (NO. 82070226).

## Conflict of Interest

The authors declare that the research was conducted in the absence of any commercial or financial relationships that could be construed as a potential conflict of interest.

## Publisher’s Note

All claims expressed in this article are solely those of the authors and do not necessarily represent those of their affiliated organizations, or those of the publisher, the editors and the reviewers. Any product that may be evaluated in this article, or claim that may be made by its manufacturer, is not guaranteed or endorsed by the publisher.
