# Supplementary material for: Circular RNA Protein Tyrosine Kinase 2 Promotes Cell Proliferation, Migration and Suppresses Apoptosis via Activating MicroRNA-638 Mediated MEK/ERK, WNT/β-Catenin Signaling Pathways in Multiple Myeloma
Source: Front Oncol. 2021 Jul 28;11:648189. doi: 10.3389/fonc.2021.648189 (PMC8355695; doi:10.3389/fonc.2021.648189)
Supplement: Supplementary file 1 [file DataSheet_1.docx]

***Supplementary Material***


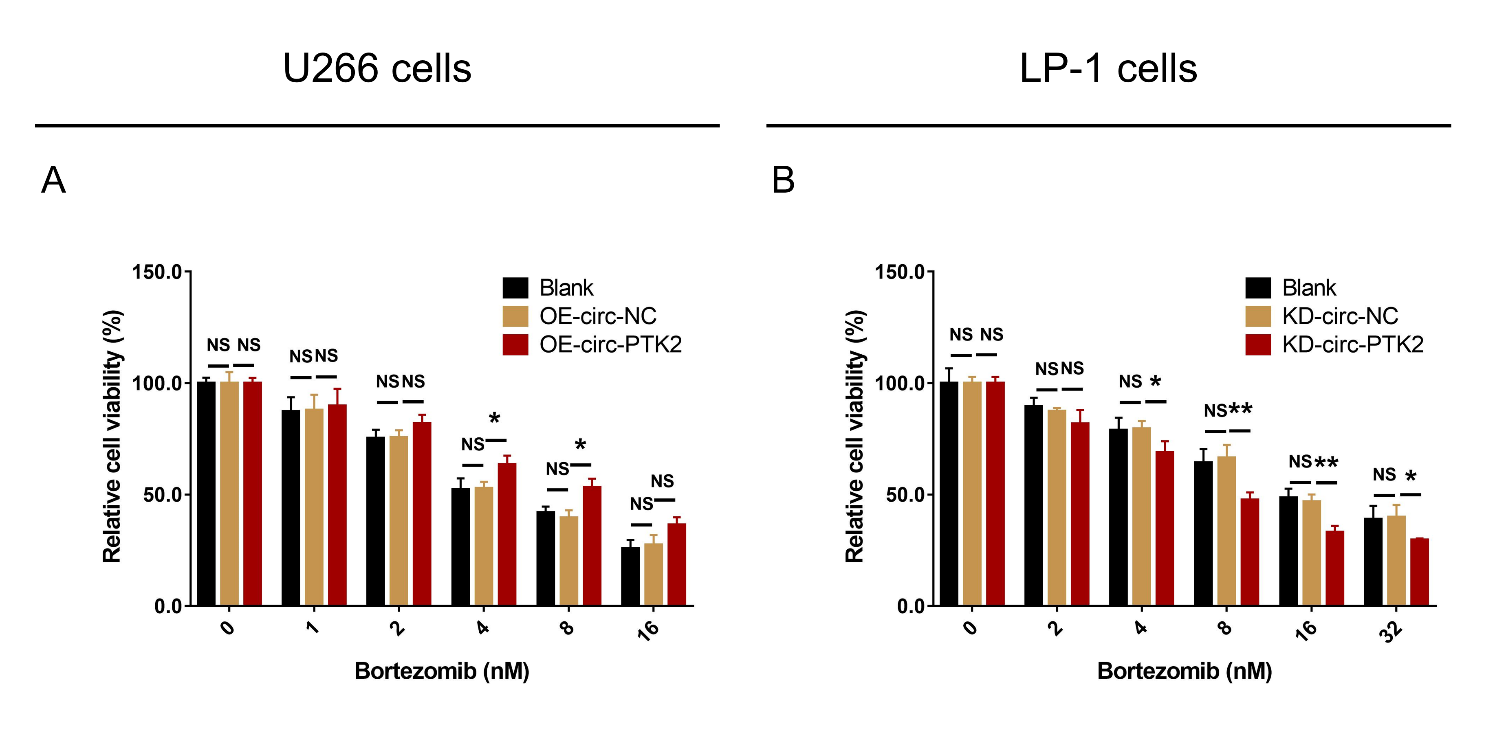


**Supplementary figure 1.** Circ-PTK2 repressed bortezomib sensitivity. Comparison of relative cell viability via CCK-8 assay under 0-16 nM bortezomib treatment among different groups of U266 cells (**A**). Comparison of relative cell viability via CCK-8 assay under 0-32 nM bortezomib treatment among different groups of LP-1 cells (**B**). Circ-PTK2, circular RNA protein tyrosine kinase 2; NS, non-significant; CCK-8, counting kit-8; OE-circ-NC, circRNA control overexpression; OE-circ-PTK2, circ-PTK2 overexpression; KD-circ-NC, circRNA control knock-down; KD-circ-PTK2, circ-PTK2 knock-down; * *P*<0.05; ** *P*<0.01. Experiments were performed in triplicates. The comparison was determined by One-way ANOVA followed by Dunnett’s multiple comparison test.
